# STUDY DESIGN OF THE AGING AND COGNITIVE HEALTH EVALUATION IN ELDERS (ACHIEVE) AND ACHIEVE-MRI STUDY

**DOI:** 10.1093/geroni/igae098.0053

**Published:** 2024-12-31

**Authors:** Alison Huang

**Affiliations:** Johns Hopkins Bloomberg School of Public Health, Baltimore, Maryland, United States

## Abstract

This presentation describes the study design of the Aging and Cognitive Health Evaluation in Elders (ACHIEVE) study and the ACHIEVE-MRI sub-study. The ACHIEVE study (n=977) is a multicenter, randomized controlled trial testing the effect of a best-practice hearing intervention vs. health education control on the primary outcome of cognitive decline over 3 years. Participants in the ACHIEVE study were 977 community-dwelling adults aged 70-84 years with untreated hearing loss and without substantial cognitive impairment. The ACHIEVE study was partially nested within the Atherosclerosis Risk in Communities [ARIC] study; participants were recruited from the ARIC study (n=238) and de novo (n=739) from the surrounding communities of four U.S. sites. Participants were randomized 1:1 to hearing intervention or health education control and followed for 3 years. ACHIEVE study participants (n=977) were median age 76.4 years, 53.5% female, 87.8% White, and 53.3% held a Bachelor’s degree or higher. The ACHIEVE-MRI study is a sub-study testing the effects of hearing intervention on 3-year change in brain MRI characteristics and cognitive function with speech-in-noise performance. The ACHIEVE-MRI study included a subset of ACHIEVE study participants (n=445) who received three-dimensional magnetic resonance imaging at baseline and at 3-year follow-up. Participants were slightly younger and had higher income and greater memory function compared to ACHIEVE study participants. The ACHIEVE-MRI study also had a larger proportion of participants recruited de novo (83%). This presentation provides important background for contextualization of ACHIEVE study and ACHIEVE-MRI study findings on brain structure and function and speech-in-noise performance presented in this symposium.

